# Supplementary material for: Positive emotion induction improves cardiovascular coping with a cognitive task
Source: PeerJ. 2021 Mar 12;9:e10904. doi: 10.7717/peerj.10904 (PMC7958892; doi:10.7717/peerj.10904)
Supplement: Supplemental Information 1 — Annex 1: Transcription of the instructions given to the participants in the positive emotional induction group. Annex 2: Transcription of the instructions given to the control group participants. [file peerj-09-10904-s001.docx]

**Annex 1 – Intervention guide for the evocation of a successful memory.**

Now you are going to do an exercise of evocation of a memory of your life, to try to concentrate on a successful experience of your life. I'm sure there's more than one, but I'd like you to think about one in particular. That moment when you felt incredibly happy and satisfied. (3”)

What you managed to do successfully. What today, when you remember it, makes you feel so proud. (3”)

During the process you may have crossed thoughts or have nothing to do with it, it is normal, just let them go and concentrate on the memory of your success. To recall it better I'm going to help you with a few questions that I'm going to ask you next, you should not answer anything, it's for you. (3”)

Try to put yourself in a comfortable position within the possible (parallel legs, comfortable back ...) trying to leave your mind calm and relaxed, open to that memory. Now, forget everything else and concentrate. Close your eyes trying to open your mind so that the successful memory of your life will find you. (3”)

Well, let's get started: Take a deep breath and try to concentrate on your memory of success. When you have it, move your hand free of electrodes as a sign that you already have it. (5”)

Try to open your mind and let that memory emerge by itself among all your thoughts. (10") (Once the subject affirms, he is asked to take another deep breath while keeping that memory in mind).

Very good. Now that you have it in mind realize by concentrating on it, a slow and deep breath. (5”)

With this memory present in your mind, try to remember more or less on what date it occurred. (15”)

Remember at what point in your life it happened. (15”)

How old you were? (10”)

What were you doing at that stage of your life? (20”)

Okay, now concentrate on the specific place where it happened. (15”)

What that place looked like.? (15”)

What was around you? (10”)

See if there are colors and sounds. (15”)

Smells (15")

If there were people with you at the time. (15”)

What ambient temperature it was? (15”)

I would like you to think about the specific action now, what it was that you did that made you feel so proud and successful? (18”)

Notice how you can feel that sensation again. (10”)

Remember how you felt (cheerful...satisfied...confident...in yourself...). (30”)

You can feel the same sensation you had when you successfully overcame that situation. (10”)

Notice how that feeling of accomplishing whatever you set out to do gradually invades your entire body. (7”)

You're regaining that great, pleasant feeling of fullness after you've done it. You've done it. (10”)

Maintain this feeling of complete satisfaction and enjoy it. (10”)

You've been able to do it, and this is the feeling I want you to focus on. (7”)

This memory is yours, is part of you and makes you great. (7”)

You were able to successfully overcome that circumstance and that makes you feel very good. (5”)

Think about that moment and notice how it makes you feel good. (8”)

This emotion that you have in this moment, thanks to that memory, makes you feel confident in yourself, happy and full of satisfaction. (5”)

Now again, by recalling this achievement you feel able to face any challenge. (7-10”)

You know you can successfully overcome anything you set your mind to. (7”)

feel this wonderful sensation a little longer. (5”)

And now, maintaining this strong feeling of well-being thanks to your achievement you can gradually open your eyes to continue with the experiment.

**Annex 2 – Distracting task for the control group: text about construction.**

During the next few minutes, I'm going to read you a text, pay close attention because then you'll have to answer a few questions about its content. A construction project is done out of necessity and others just for fun, but anyway, the best builders around the world think big. They use sophisticated tools, the best materials, high technology and innovation and finally creativity.

Today's construction projects can often go higher and last longer than ever before. Mega constructions are produced all over the world, in Asia, the United States, Europe, Africa, South America and Canada. From huge bridges, tunnels designed to relieve urban traffic congestion to sophisticated buildings or underwater dive sites. Examples include the world's largest roller coaster, the Channel or the Palm Island of Dubai

Construction is a risky job that is full of unforeseen challenges and extreme tests of planning and strength. When Hurricane Katrina struck New Orleans, it was a big impact on this population. Because the entire city had to be rebuilt. Including thousands of homes, that's when mega-builders came up with the brilliant idea of creating hurricane-proof safe houses. But... Creating hurricane-proof houses is a very difficult task. (5”)

Any construction process requires thinking of the best materials, the most resistant, as well as a good job of architectural planning, engineering and above all innovation and creativity. (5”)

When the idea of building safe houses that are hurricane-proof or can withstand attacks in extreme environmental conditions was raised, the people of New Orleans put this idea on rest. It is an area of frequent passage of hurricanes, storms and strong winds. Although they are used to losing their homes and rebuilding them, it meant a change of mentality and an improvement in the quality of life. (5-7”)

Willy Tomas, one of the best civil engineers in the United States, came up with an innovative and brilliant idea to build pre-fabricated houses as if they were cars. It's about copying the idea Henry Ford came up with in 1901 in building cars through assembly lines. What by then revolutionized the industrial organization called Taylorism that has deepened in the twenty-first century. (5-7”)

Taylorism is the application of scientific methods with a positivist and mechanistic orientation to the study of the relationship between the worker and modern industrial production techniques, in order to maximize the efficiency of labor, machines and tools, through the systematic division of tasks, the rational organization of work in its sequences and processes, and the timing of operations, plus a system of motivation through the payment of performance bonuses, suppressing all improvisation in industrial activity. (5”)

Frederick Taylor attempted to eliminate unnecessary worker movements with the desire to maximize the productive potential of the industry. He did a study with the objective of eliminating useless movements and establishing by means of chronometers the time necessary to carry out each specific task. (5-7”)

Taylor's system lowered production costs because less wages had to be paid, companies even paid less money for each piece to get workers to hurry. For this system to work properly, it was essential that the workers were supervised, and so a special group of employees emerged, who were in charge of supervising, organizing, and directing the work. (7”)

His obsession with productive time led him to work on the concept of a chronometer in the productive process, an idea that would surpass that of a workshop, typical of the first phase of the Industrial Revolution. (7”)

The division of labor proposed by Taylor effectively reduces costs and scientifically reorganizes labor, but finds a growing rejection of the proletariat, an element that added to the crisis of structural expansion of the market (speed of circulation of goods) would lead to a practical reformulation in the twentieth century which is the idea of Fordism. (7”)

Willy Tomas, one of the best civil engineers in the United States, came up with an innovative and brilliant idea of building pre-fabricated houses as if they were cars, copying Fordism and with the technology of specific products so that these houses can withstand extreme weather conditions as could be the case in a hurricane.( 7")

architects and raised them to carry out this brilliant idea with funding from the central government of the United States. (7”)

Once the great engineers and architects had gathered and after a civil engineering work, where they investigated the best way for a house to withstand attacks from extreme environmental conditions such as hurricanes, they innovated applying this brilliant idea already thought by Henry Ford more than a century ago. (7”)

They created a matrix house divided into six modules, considering the form, design and type of material. These would be assembled one by one on an assembly line, as if they were the parts that make up an automobile and once finished, each part would be joined to create the final house. (7”)

Let us not forget that for the construction of these materials each one of them would go through rigorous quality systems. (7”)

Once the final house was built, these would be stored in panels ready to be shipped to each area where the hurricane had passed. (7”)

This type of ideas has innovated the mega-construction and engineering market, since in a country like the United States, where this type of phenomena is common, it means a saving of billions of dollars. (7”).

Building pre-fabricated houses as if it were an assembly of cars, lowers costs and increases the speed of construction. Safe, innovative and high-tech houses can be brought as quickly and economically as possible. This is one more advance of the human being in the adaptation of the coexistence of this one to natural phenomena so difficult to palliate their effects. (7”)

Now take a few seconds to try to remember the text and continue the experiment.
