# Supplementary material for: Positive emotion induction improves cardiovascular coping with a cognitive task
Source: PeerJ. 2021 Mar 12;9:e10904. doi: 10.7717/peerj.10904 (PMC7958892; doi:10.7717/peerj.10904)
Supplement: Supplemental Information 2 — Anexo 1: Transcripción de las instrucciones dadas a los participantes del grupo de inducción emocional positiva. Anexo 2: Transcripción de las instrucciones dadas a los participantes del grupo control. [file peerj-09-10904-s002.docx]

**Anexo 1**

**Intervención para el GRUPO CONTROL (GC):**

“Durante los próximos minutos, te voy a leer un texto, presta mucha atención porque después tendrás que responder a unas preguntas sobre su contenido. Un proyecto de construcción se realiza por necesidad y otras sólo por diversión, pero de cualquier manera, los mejores constructores en todo el mundo piensan a lo grande. Utilizan sofisticadas herramientas, los mejores materiales, la alta tecnología e innovación y por último la creatividad.

Es muy frecuente que los proyectos de construcción actuales puedan llegar más alto y resistir más que nunca. Se producen mega construcciones en todo el mundo, tanto en Asia, Estados Unidos, Europa, África, Sudamérica y Canadá. Desde enormes puentes, túneles diseñados para aliviar la congestión del tráfico urbano a sofisticados edificios o sitios submarinos de buceo. Ejemplo de ello, son la montaña rusa más grande del mundo, el canal de la Mancha o la isla Palmera de Dubái.

La construcción es un trabajo arriesgado que está lleno de desafíos imprevistos y pruebas extremas de planificación y fortaleza. Cuando ocurrió el huracán Katrina en Nueva Orleans, fue un impacto grande para esta población. Porque se tuvo que volver a reconstruir toda la ciudad. Incluyendo miles de casas, es entonces cuando los mega constructores llegaron a la brillante idea de crear casas seguras a prueba de huracanes. Pero... Crear casas seguras a prueba de huracanes es tarea muy difícil. (5”).

Todo proceso de construcción requiere pensar en los mejores materiales, los más resistentes, como también, en un buen trabajo de planificación de arquitectura, ingeniería y sobre todo innovación y creatividad. (5”).

Cuando se planteó la idea de construir casas seguras a prueba de huracanes o que soporten ataques en condiciones medio ambientales extremas, a la población de Nueva Orleans calló muy bien esta idea. Se trata de una zona de paso habitual de huracanes, tormentas y fuertes vientos. Aunque están acostumbrados a perder sus casas y volver a construirlas, suponía un cambio de mentalidad y mejora de la calidad de vida. (5-7”).

Willy Tomas uno de los mejores ingenieros civiles de Estados Unidos, se le ocurrió una idea innovadora y brillante de construir casas pre-fabricadas como si de coches fueran. Se trata de copiar la idea que Henry Ford ideó en 1901 en la construcción de coches a través de cadenas de montajes. Lo que para entonces revolución la organización industrial llamándose Taylorismo que se ha profundizado en el [siglo XXI](https://es.wikipedia.org/wiki/Siglo_XXI). (5-7”).

Se conoce como “Taylorismo” a la aplicación de [métodos científicos](https://es.wikipedia.org/wiki/M%C3%A9todo_cient%C3%ADfico) de orientación positivista y mecanicista al estudio de la relación entre el [obrero](https://es.wikipedia.org/wiki/Construcci%C3%B3n) y las técnicas modernas de producción industrial, con el fin de maximizar la eficiencia de la [mano de obra](https://es.wikipedia.org/wiki/Mano_de_obra), máquinas y herramientas, mediante la división sistemática de las tareas, la organización racional del [trabajo](https://es.wikipedia.org/wiki/Trabajo) en sus secuencias y procesos, y el cronometraje de las operaciones, más un sistema de [motivación](https://es.wikipedia.org/wiki/Motivaci%C3%B3n) mediante el pago de primas al rendimiento, suprimiendo toda improvisación en la [actividad industrial](https://es.wikipedia.org/w/index.php?title=Actividad_industrial&action=edit&redlink=1).(5”).

[Frederick Taylor](https://es.wikipedia.org/wiki/Frederick_W._Taylor) intentó eliminar por completo los movimientos innecesarios de los obreros con el deseo de aprovechar al máximo el potencial productivo de la [industria](https://es.wikipedia.org/wiki/Industria). Hizo un estudio con el objetivo de eliminar los movimientos inútiles y establecer por medio de cronómetros el tiempo necesario para realizar cada tarea específica. (5-7”).

El sistema de Taylor bajó los costos de producción porque se tenían que pagar menos salarios, las empresas incluso llegaron a pagar menos dinero por cada pieza para que los [obreros](https://es.wikipedia.org/wiki/Construcci%C3%B3n) se diesen más prisa. Para que este sistema funcione correctamente era imprescindible que los trabajadores estuvieran supervisados y así surgió un grupo especial de empleados, que se encargaba de la supervisión, organización y dirección del trabajo. (7”).

Su obsesión por el tiempo productivo lo llevó a trabajar el concepto de cronómetro en el proceso productivo, idea que superaría a la de taller, propia de la primera fase de la [Revolución Industrial](https://es.wikipedia.org/wiki/Revoluci%C3%B3n_Industrial). (7”).

La división del trabajo planteada por Taylor efectivamente reduce los costos y reorganiza científicamente el trabajo, pero encuentra un rechazo creciente del [proletariado](https://es.wikipedia.org/wiki/Proletariado), elemento que sumado a la crisis de expansión estructural de mercado (por velocidad de circulación de la mercancía) lo llevaría a una reformulación práctica en el [siglo XX](https://es.wikipedia.org/wiki/Siglo_XX) que es la idea de [fordismo](https://es.wikipedia.org/wiki/Fordismo). (7”).

Pero volviendo al tema que hemos introducido… Willy Tomas uno de los mejores ingenieros civiles de Estados Unidos, se le ocurrió una idea innovadora y brillante de construir casas pre-fabricadas como si de coches fueran, copiando el fordismo y con la tecnología de productos específicos para que esas casas puedan soportar condiciones climática extremas como podría darse el caso en un huracán.( 7”).

Para ello Willy Tomas, llamó a los mejores ingenieros y arquitectos y les planteó llevar a cabo estar brillante idea con la financiación del Gobierno central de los Estados Unidos. (7”).

Una vez reunidos los grandes ingenieros y arquitectos y después de un trabajo de ingeniería civil, donde se investigó la mejor manera que una casa pueda soportar ataques de condiciones medio ambientales extremas como los huracanes, se innovó aplicando esta genial idea ya pensada por Henry Ford hace más de un siglo. (7”).

Crearon una matriz de casa dividida en seis módulos, teniendo en cuenta la forma, diseño y tipo de material. Éstos se irían montando de uno en uno en una cadena de montaje, como si fueran las partes que forman un automóvil y una vez finalizado, se uniría cada parte para crear la casa final. (7”).

No olvidemos que para la construcción de estos materiales cada uno de ellos pasaría por rigurosos sistemas de calidad. (7”).

Una vez hecha la casa final, éstas se almacenarían en paneles listas para ser enviadas a cada zona donde hubiese pasado el huracán. (7”).

Este tipo de ideas ha innovado el mercado de la mega construcción e ingeniería, ya que en un país como en Estados Unidos, donde es habitual este tipo de fenómenos supone un ahorro de miles de millones de dólares. (7”).

Construir casas prefabricadas como si fuera un montaje de coches, abarata gastos y aumenta la rapidez de la construcción. Pudiéndose llevar casas seguras, innovadoras y de alta tecnología con la mayor rapidez y ahorro económico posible. Esto es un avance más del ser humano en la adecuación de la convivencia de éste a fenómenos naturales tan difíciles de paliar sus efectos. (7”).

Ahora tómate unos segundos para intentar recordar el texto y continuar con el experimento.”

**6.2. Anexo 2.**

**Intervención para GRUPO EXPERIMENTAL :**

“Ahora vas a hacer un ejercicio de evocación de un recuerdo de tu vida, para intentar concentrarte en una experiencia de éxito de tu vida. Seguro que hay más de una, pero me gustaría que pensaras en una en concreto. Ese momento en el que te has sentido increíblemente alegre y satisfecho. (3´´)

Eso que lograste hacer con éxito, de lo que hoy cuando lo recuerdas hace que te sientas tan orgulloso/a. (3´´).

Durante el proceso puede que te aparezcan pensamientos cruzados o que no tengan nada que ver, es normal, simplemente déjalos marchar y concéntrate en el recuerdo de tu éxito. Para recordarlo mejor voy a ayudarte mediante unas preguntas que voy a realizarte a continuación, no debes contestar nada, es para ti.

Te recomiendo que utilices el recuerdo que has pensado al comenzar el experimento, aunque si aparece otro nuevo que sea importante para ti puedes quedarte con él. (3´´).

Intenta ponerte en una posición cómoda dentro de lo posible (piernas paralelas, espalda cómoda…) intentando dejar tu mente tranquila y relajada, abierta a ese recuerdo. Ahora, olvida todo lo demás y concéntrate. Cierra tus ojos intentando abrir tu mente para que ese recuerdo de éxito de tu vida, te encuentre. (3´´).

Bien, vamos a comenzar:

Haz una respiración profunda e intenta concentrarte en tu recuerdo de éxito. Cuando lo tengas, mueve tu mano libre de electrodos como señal de que ya lo tienes. (5´´) Intenta abrir tu mente y deja que ese recuerdo emerja por si sólo entre todos tus pensamientos. (10 ´´). (Una vez que el sujeto afirma se le pide que realice otra respiración profunda mientras mantiene ese recuerdo en mente).

Muy bien.

Ahora que lo tienes en mente realiza concentrándote en él, una respiración lenta y profunda. (5´´).

Con este recuerdo presente en tu mente, intenta recordar más o menos en qué fecha ocurrió (15´´).

Recuerda en qué momento de tu vida sucedió (15´´).

Cuántos años tenías (10´´).

Qué estabas haciendo en esa etapa de tu vida (20´´).

Bien, ahora concéntrate en el lugar concreto dónde ocurrió… (15´´).

Cómo era ese lugar (15´´).

Qué había a tu alrededor (10´´).

Fíjate si hay colores y sonidos (15´´).

Olores (15´´).

Si había personas contigo en ese momento (15´´).

Qué temperatura ambiente hacía (15´´).

Me gustaría ahora que pensarás en la acción en concreto…qué fue eso que hiciste que te hizo sentir tan orgulloso y exitoso... (18´´).

Nota cómo eres capaz de volver a sentir esa sensación (10´´).

Recuerda cómo te sentiste (alegre…satisfecho…confiado en ti mismo…) (30´´).

Eres capaz de sentir la misma sensación que tuviste cuando lograste superar con éxito aquella situación (10´´).

Nota cómo esa sensación de lograr cualquier cosa que te propongas, va invadiendo poco a poco todo tu cuerpo (7´´).

Estas volviendo a notar esa gran y agradable sensación de plenitud tras haberlo logrado. Tú lo has conseguido. (10´´).

Mantén esta sensación de satisfacción plena y disfrútala. (10´´).

Tú has sido capaz de lograrlo y esta es la sensación en la que quiero que te concentres. (7´´).

Este recuerdo es tuyo, forma parte de ti y te engrandece. (7´´).

Fuiste capaz de lograr superar con éxito aquella circunstancia y eso hace que te sientas muy bien (5´´).

Piensa en aquel momento y nota como te hace sentir bien (8´´).

Está emoción que tú tienes en este momento, gracias a ese recuerdo, te hace sentir con confianza en ti mismo, alegre y lleno de satisfacción. (5´´).

Ahora de nuevo, al recordar este logro te sientes capaz de afrontar cualquier reto (7-10´´).

Sabes que puedes lograr superar con éxito cualquier cosa que te propongas. (7´´).

Quédate sintiendo esta sensación maravillosa un poco más... (5´´).

Y ahora, manteniendo esta sensación tan fuerte de bienestar gracias a tu logro puedes ir abriendo poco a poco tus ojos para continuar con el experimento.”.
